# Therapeutic Potential of Emricasan, a Pan-Caspase Inhibitor, in Reducing Cell Death and Extracellular Matrix Accumulation in Fuchs Endothelial Corneal Dystrophy

**DOI:** 10.3390/cells14070498

**Published:** 2025-03-27

**Authors:** Sohya Fujimoto, Mako Endo, Shigehito Tonomura, Fuuga Tsuji, Hirotaka Haraguchi, Kanna Hasegawa, Taisuke Numao, Ayaka Izumi, Theofilos Tourtas, Ursula Schlötzer-Schrehardt, Friedrich Kruse, Yuki Oyama, Masahito Ikawa, Albert S. Jun, Noriko Koizumi, Naoki Okumura

**Affiliations:** 1 Department of Biomedical Engineering, Faculty of Life and Medical Sciences, Doshisha University, Kyotanabe 602-8580, Japan; 2 ActualEyes Inc., Kyotanabe 610-0343, Japan; 3 Department of Ophthalmology, University of Erlangen-Nürnberg, 430074 Erlangen, Germany; 4 Graduate School of Pharmaceutical Sciences, Osaka University, Suita 565-0871, Japan; 5 Department of Experimental Genome Research, Research Institute for Microbial Diseases, Osaka University, Suita 565-0871, Japan; 6 Department of Ophthalmology, University of Virginia School of Medicine, Charlottesville, VA 22903, USA

**Keywords:** Fuchs endothelial corneal dystrophy, caspase inhibitor, apoptosis, extracellular matrix, corneal endothelial cells, animal models

## Abstract

Fuchs endothelial corneal dystrophy (FECD) is a progressive disorder characterized by endothelial cell loss and excessive extracellular matrix (ECM) accumulation leading to corneal dysfunction. Emricasan, a pan-caspase inhibitor, was investigated for its therapeutic potential in suppressing these pathological changes. Patient-derived FECD cells and stress-induced cell models were treated with emricasan to assess its effects on apoptosis and ECM production. Caspase-specific knockdown experiments were performed to identify key mediators. *Col8a2*^Q455K/Q455K^ mice, model mice of early-onset FECD, received twice-daily administration of 0.1% emricasan eye drops from 8 to 28 weeks of age. Endothelial cell density, hexagonality, cell size variation, and guttae area were evaluated by contact specular microscopy, while transcriptomic changes were analyzed via RNA sequencing. Emricasan effectively reduced apoptosis and ECM production in vitro by selectively inhibiting caspase-7 without affecting canonical TGF-β signaling. In vivo, emricasan-treated mice exhibited significantly higher endothelial cell density, improved hexagonality, and reduced variation in cell size compared with controls. Transcriptome analysis revealed distinct gene expression changes in the corneal endothelium following emricasan treatment. These findings suggest that emricasan exerts dual protective effects by inhibiting caspase-7-mediated ECM accumulation and broadly suppressing apoptosis, highlighting its potential as a pharmacological therapy for FECD.

## 1. Introduction

Fuchs endothelial corneal dystrophy (FECD) is a progressive eye disease characterized by the formation of guttae, excrescences of Descemet membrane, and corneal endothelial cell degeneration [1,2,3]. The disease typically begins with the formation of central guttae, which, as they become more confluent, cause light scatter and consequent visual impairment [4,5]. As the disease progresses, corneal endothelial dysfunction advances until the remaining endothelial cells can no longer maintain corneal deturgescence, resulting in corneal edema and severe vision loss [1,2,3]. While corneal transplantation remains the standard treatment for FECD, several challenges persist, including a global donor cornea shortage (particularly outside the United States), immune rejection, and long-term graft failure due to continued endothelial cell loss [6,7,8,9,10]. These limitations underscore the urgent need for alternative therapeutic approaches, particularly pharmacological interventions.

Various molecular pathways contribute to FECD pathogenesis, with apoptotic cell death representing a central mechanism supported by substantial evidence [3]. Corneal endothelial cells (CECs) in FECD patients demonstrate enhanced vulnerability to apoptosis triggered by oxidative stress compared with endothelial cells from healthy controls [11,12]. This susceptibility stems from multiple factors, including accumulated oxidative damage to DNA (particularly affecting mitochondrial DNA) and compromised antioxidant defense systems [11]. Cell death in FECD is further promoted by chronic activation of the unfolded protein response pathway [13,14,15]. The disease progression is accelerated by dysfunctional mitochondria and defective mitophagy mechanisms [16]. Moreover, the characteristic guttae formations trigger apoptotic cascades in adjacent CECs, establishing a self-perpetuating cycle of cellular degeneration [17]. These insights into FECD pathogenesis, particularly the pivotal role of apoptosis in disease progression, suggest that targeting apoptotic pathways could offer a promising therapeutic approach for FECD.

Apoptosis constitutes a fundamental cellular mechanism essential for tissue homeostasis, with its dysregulation contributing to numerous pathological conditions [18,19]. This regulated cell death process involves intricate molecular pathways, primarily mediated through caspase activation and Bcl-2 family proteins, where both excessive and insufficient activation can result in disease states including autoimmune disorders, neurodegenerative diseases, and cancer [18,20,21,22,23]. Contemporary therapeutic approaches targeting apoptotic pathways have yielded promising outcomes, particularly through the development of small-molecule inhibitors targeting antiapoptotic proteins such as BCL-2, IAPs, and MDM2 [24,25,26,27,28]. Based on this scientific rationale, we sought to investigate the therapeutic potential of compounds specifically targeting apoptotic pathways as FECD treatments.

In the current study, we used various cell death-induced FECD cellular models to investigate whether emricasan, a pan-caspase inhibitor that has been evaluated in multiple clinical trials for non-ophthalmic conditions [29,30,31,32,33], could suppress cell death in FECD. Furthermore, we explored how pan-caspase inhibition affects cell death as well as pathological extracellular matrix (ECM) production. Through selective caspase knockdown using siRNA, we identified specific caspases involved in ECM production and elucidated their underlying mechanisms. Finally, we evaluated the therapeutic potential of topical emricasan administration using an FECD mouse model.

## 2. Materials and Methods

### 2.1. Ethics Statement

All human tissue specimens were handled in accordance with the ethical principles of the Declaration of Helsinki. The study protocol received approval from the Ethics Committee of Friedrich-Alexander University Erlangen-Nürnberg (FAU; approval no. 140_20 B, approved date: 30 March 2018) and the Ethics Committee for Scientific Research at Doshisha University (approval no. 20009, approved date: 16 September 2020). Descemet membranes with CECs were obtained from FECD patients undergoing Descemet membrane endothelial keratoplasty (DMEK) at the University of Erlangen-Nürnberg, after obtaining written informed consent from the patients for the surgical procedure and the use of surgical specimens for research purposes.

### 2.2. Animals

All animal experiments in this study were approved by the Animal Care and Use Committees of Doshisha University (approval number: Doshisha-A-20030, approval date: 1 April 2020), and were conducted in accordance with institutional guidelines and regulations. All procedures adhered to the ARVO Statement for the Use of Animals in Ophthalmic and Vision Research. C57BL/6J mice were obtained from CLEA Japan, Inc. (Tokyo, Japan). The *Col8a2*^Q455K/Q455K^ mice were used as a model for FECD, as previously described [34,35]. Slc:JW/CSK rabbits were purchased from Shimizu Laboratory Supplies (Kyoto, Japan).

### 2.3. Cell Culture and Establishment of Cell Lines

CECs were obtained from normal human and FECD donor corneas. The FECD patients demonstrated ≥50 CTG triplet repeat expansions in the *TCF4* gene, as confirmed by genomic DNA analysis of peripheral blood samples using established methods [36].

Primary CEC cultures were established following a published protocol [37,38]. Briefly, the Descemet membrane with corneal endothelium was enzymatically digested in OptiMEM-I (Life Technologies Corp., Carlsbad, CA, USA) containing 1 mg/mL collagenase A (Roche Applied Science, Penzberg, Germany). Isolated HCECs were washed and seeded on 24-well plates (1.9 cm^2^/well) coated with laminin E8 fragment (iMatrix-511; Nippi, Inc., Tokyo, Japan). Cells were maintained in culture medium consisting of OptiMEM-I supplemented with 8% fetal bovine serum (FBS), 10 μM Y-27632 (Wako Pure Chemical Industries, Ltd., Osaka, Japan) (first 24 h only), 10 μM SB203580 (Wako Pure Chemical Industries, Ltd.), 1 μM SB431542 (Wako Pure Chemical Industries, Ltd.), 5 ng/mL epidermal growth factor (Life Technologies Corp.), 20 μg/mL ascorbic acid (Sigma-Aldrich, Burlington, MA, USA), 200 mg/L calcium chloride (Sigma-Aldrich), 0.08% chondroitin sulfate (Sigma-Aldrich), and 50 μg/mL gentamicin (Life Technologies Corp.).

Primary HCECs were cultured for 14–21 days before immortalization via lentiviral transduction with vectors encoding SV40 large T antigen and human telomerase reverse transcriptase (hTERT), following an established protocol [39]. The gene-coding sequences were PCR-amplified, TA-cloned into lentiviral vectors, and co-transfected with helper plasmids (pLP1, pLP2, and pLP/VSVG) into HEK293T cells (RCB2202; Riken Bioresource Center, Ibaraki, Japan). Viral supernatants were collected 48 h post-transfection and used to transduce HCECs in the presence of polybrene. The resulting immortalized cell lines were maintained in Dulbecco’s modified Eagle’s medium (DMEM; Life Technologies Corp.) supplemented with 10% fetal bovine serum and 1% penicillin–streptomycin (Nacalai Tesque, Kyoto, Japan). The cells were passaged using 0.05% trypsin–EDTA (Life Technologies Corp.) when reaching 80% confluency. Immortalized cell lines were designated as iHCEC (from normal human) and iFECD (from FECD patients with ≥50 repeats).

To investigate the effects of emricasan (ChemsceneLLS, Monmouth Junction, NJ, USA) on TGF-β-induced cell death and ECM accumulation, iHCEC and iFECD cells were treated with 10 ng/mL TGF-β2 (Wako Pure Chemical Industries, Ltd.) for 24 h following established protocols. Emricasan (10 μM) effects were evaluated by supplementing the culture medium during TGF-β2 treatment. Other pan-caspase inhibitors, including Z-VAD-FMK (Peptide Institute, Osaka, Japan), Z-VD-FMK (Wako Pure Chemical Industries, Ltd.), and nivocasan (Adooq Bioscience, Irvine, CA, USA) (all at 10 μM), were used as controls. To evaluate the protective effects of emricasan against various stressors, iHCEC cells were exposed to the following multiple stress conditions for 24 h: UV radiation (100 mJ/cm^2^), proteasome inhibitor MG132 (20 μM; Enzo Life Science, Farmingdale, NY, USA), the ER stress inducer thapsigargin (10 μM; Wako Pure Chemical Industries, Ltd.), the protein kinase inhibitor staurosporine (STP) (0.5 μM; Sigma-Aldrich Corp., St. Louis, MO, USA), or the protein synthesis inhibitor anisomycin (0.1 μM; Wako Pure Chemical Industries, Ltd.), to establish multiple in vitro FECD models.

### 2.4. Annexin V Assay

Cultured cells were washed twice with phosphate-buffered saline (PBS), followed by enzymatic dissociation using Accutase^®^ (Innovative Cell Technologies, San Diego, CA, USA) at 37 °C for 5 min. The cell suspension was collected and processed for apoptosis analysis. Apoptotic cells were detected by Annexin V staining using the MEBCYTO^®^ Apoptosis Kit (Medical & Biological Laboratories Co., Ltd., Nagoya, Japan) according to the manufacturer’s protocol. The proportion of Annexin V-positive cells was quantified using an SH800S Cell Sorter (Sony Biotechnology, San Jose, CA, USA). Each experimental condition was assessed using three samples.

### 2.5. Caspase 3/7 Activity Assay

iFECD cells were seeded in 96-well plates at a density of 1.0 × 10⁴ cells/cm^2^ and cultured for 48 h. The cells were then exposed to TGF-β2 (10 ng/mL) in DMEM for 24 h to induce apoptotic responses. Caspase 3/7 activity was quantified using the Caspase-Glo^®^ 3/7 Assay System (Promega, Madison, WI, USA) according to the manufacturer’s instructions. Luminescence was measured using a Veritas™ Microplate Luminometer (Promega). Each experimental condition was assessed using six samples.

### 2.6. Immunoblotting

Cell lysates were prepared by washing cultured cells with ice-cold PBS followed by lysis in radioimmunoprecipitation assay (RIPA) buffer supplemented with phosphatase inhibitor cocktail 2 (Sigma-Aldrich) and protease inhibitor cocktail (Roche Applied Science). After centrifugation to remove cellular debris, proteins in the supernatant were separated by sodium dodecyl sulfate–polyacrylamide gel electrophoresis (SDS-PAGE) and transferred to polyvinylidene difluoride (PVDF) membranes. Membranes were blocked with 3% nonfat dry milk and incubated overnight at 4 °C with primary antibodies. Primary antibodies from Cell Signaling Technology (Danvers, MA, USA) included apoptotic markers (caspase-1, -3, -4, -7, -8, -9, and PARP; all at 1:1000), TGF-β signaling markers (Smad2 at 1:1000 and phospho-Smad2 at 1:500), and the EMT marker Snail1 (1:1000). Additional primary antibodies included fibronectin (1:15,000; Becton, Dickinson and Company, Franklin Lakes, NJ, USA) and GAPDH (1:3000; Medical & Biological Laboratories Co., Ltd., Nagoya, Japan). Proteins were detected using horseradish peroxidase-conjugated secondary antibodies (1:5000; GE Healthcare, Piscataway, NJ, USA) and enhanced chemiluminescence with the ECL Advanced Western Blotting Detection Kit (Nacalai Tesque). For all comparative analyses, experimental samples and their respective controls were run on the same gel/blot to ensure accurate comparison. Image adjustments (brightness and contrast) were applied uniformly to entire blots and were minimal to preserve data integrity. Cropped blot images presented in figures retain all relevant bands of interest and information necessary for interpretation.

### 2.7. Immunofluorescence Staining

Cells were fixed with 4% paraformaldehyde (PFA; Nacalai Tesque) at room temperature for 10 min on glass slides. Excess PFA was removed by washing with PBS, and the samples were permeabilized with 0.5% Triton X-100 (Nacalai Tesque). To block nonspecific binding, the samples were incubated with 1% bovine serum albumin (Thermo Fisher Scientific, Waltham, MA, USA). Primary antibodies against fibronectin (1:200; BD Biosciences, San Jose, CA, USA) and collagen I (1:200; Merck Millipore, Darmstadt, Germany) were applied to the samples. Secondary detection was performed using Alexa Fluor 488-conjugated goat antimouse antibodies (Thermo Fisher Scientific) at a 1:1000 dilution. Nuclear counterstaining was conducted using 4′,6-diamidino-2-phenylindole (DAPI; Vector Laboratories, Burlingame, CA, USA). The stained slides were examined under a fluorescence microscope (DM 2500; Leica Microsystems, Wetzlar, Germany).

### 2.8. qPCR Analysis

Total RNA was extracted from cells using the RNeasy Mini Kit (QIAGEN, Hilden, Germany) according to the manufacturer’s protocol. Cell lysis was accomplished by adding 350 µL of Buffer RLT per well and scraping the cells with a cell scraper. The resulting lysate was mixed with 70% ethanol and transferred to a RNeasy Mini Spin Column, followed by centrifugation at 10,000× *g* for 15 s. An initial wash with Buffer RW1 was followed by DNA contamination removal using RQ1 RNase-Free DNase (Promega), which was incubated at room temperature for 20 min. Additional washing cycles with Buffer RW1 and Buffer RPE were performed, with a final centrifugation at 14,000× *g* for 2 min. The purified RNA was then eluted with RNase-free water and quantified using a NanoDrop spectrophotometer (Thermo Fisher Scientific Inc.). 

The cDNA was synthesized using 100 ng of total RNA in a carefully prepared 20 µL reaction mixture. The mixture contained 5× RT Buffer, 10 mM dNTPs, Random Primer, ReverTra Ace (TOYOBO, Osaka, Japan), and RNase Inhibitor. The thermal cycling process included three critical temperature stages: an initial annealing at 30 °C for 10 min, followed by extension at 42 °C for 1 min, and a final denaturation at 99 °C for 5 min. Gene expression levels were analyzed using TaqMan^®^ real-time PCR (Applied Biosystems, Foster City, CA, USA). The TaqMan primers used were FN1 (Hs00365052_ml), COL1A1 (Hs00164004_ml), SNAIL1 (Hs00195591_ml), SNAIL2 (Hs00195591_ml), and GAPDH (Applied Biosystems). Real-time PCR was conducted using the StepOne (Applied Biosystems) system, with GAPDH serving as the internal standard.

### 2.9. siRNA

To investigate the potential suppression of ECM through targeted caspase inhibition, we designed a comprehensive siRNA-mediated knockdown experiment targeting caspase-1, -3, -4, -7, -8, and -9. The study utilized induced iFECD cells, which were seeded at a density of 5 × 10^4^ cells per well in 12-well plates and cultured for 24 h. After washing twice with PBS, we prepared siRNA mixtures: Lipofectamine^®^ RNAiMAX Reagent (Invitrogen, Tokyo, Japan) mixed with Opti-MEM and individual siRNAs with the following final concentrations: siCASP1 at 1 pmol/μL, siCASP3 at 10 pmol/μL, siCASP4 at 1 pmol/μL, siCASP7 at 10 pmol/μL, siCASP8 at 10 pmol/μL, and siCASP9 at 10 pmol/μL (Silencer^®^ Select siRNAs, Invitrogen/Ambion, Tokyo, Japan), along with a Silencer™ Negative Control siRNA. The Lipofectamine solution and each siRNA solution were mixed in a 1:1 ratio and incubated for 5 min. Cells were divided into two groups: a nontargeting (NT) siRNA control group and specific caspase siRNA knockdown groups. After transfection for 24 h, the medium was replaced, and the cells were cultured for an additional 24 h. Subsequently, the cells were exposed to TGF-β2 (10 ng/mL) in DMEM for 24 h.

### 2.10. Preparation of Emricasan Eye Drops

Emricasan (15 mg) was dissolved in 100 µL of 99% ethanol (Nacalai Tesque). After complete homogenization of the solution using a vortex mixer, the ethanol was completely evaporated under nitrogen gas. Subsequently, 15 µL of polysorbate 80 (Sigma Aldrich) as a surfactant and 30 µL of 1% glycerin (Kenei Pharmaceutical, Osaka, Japan), as an isotonizing agent, were added and thoroughly mixed. To this mixture, 2.94 mL of 0.2% phosphate buffer was added, and the mixture was completely homogenized using a vortex mixer. Finally, the pH was adjusted to 7.0 using diluted hydrochloric acid and sodium hydroxide (both from Nacalai Tesque) to prepare the emricasan eye drops.

### 2.11. High-Performance Liquid Chromatography

Slc:JW/CSK rabbits (n = 3) received a single topical administration of 50 µL of 0.1% emricasan eye drops in their right eyes. At 1 h after administration, the animals were euthanized and their eyes were enucleated. The corneal tissue was harvested and mechanically separated into epithelial, stromal, and endothelial layers, and each layer was weighed separately. Individual tissue samples were homogenized in 2 mL of acetonitrile (Nacalai Tesque) using a glass homogenizer (Tokyo Garasu Kikai Ltd., Tokyo, Japan). The resulting suspensions were transferred to glass test tubes and centrifuged at 3000 rpm for 10 min at 4 °C (Tomy Seiko Ltd., Tokyo, Japan). The supernatant was collected, and the precipitate was re-extracted with 1 mL of acetonitrile, followed by centrifugation. The combined supernatants were evaporated under nitrogen gas and reconstituted in 200 µL of acetonitrile for concentration analysis.

Drug concentrations were determined using high-performance liquid chromatography (HPLC; Shimadzu Corp., Kyoto, Japan) under the following conditions: mobile phase consisted of acetonitrile containing 0.05% trifluoroacetic acid (Wako Pure Chemical Industries, Ltd.) and water (60:40 *v*/*v*); column temperature was maintained at 40 °C; flow rate was set at 1.0 mL/min; detection wavelength was 254 nm; injection volume was 10 µL; and analysis time was 10 min. Tissue drug concentrations were calculated by dividing the total drug amount by the weight of each tissue. Assuming a specific gravity of approximately 1, tissue weights (g) were converted to volumes (mL) for all corneal layers during the concentration calculations.

### 2.12. Contact Specular Microscopy Imaging in Col8a2^Q455K/Q455K^ Mice

A 2 µL volume of 0.1% emricasan eye drops was administered twice daily in the right eye of *Col8a2*^Q455K/Q455K^ mice from 8 to 28 weeks of age (Emricasan group, n = 68). The control group received vehicle eye drops without emricasan following the same procedure (vehicle group, n = 35). Under general anesthesia, 2 µL of Benoxyl ophthalmic solution (Santen Pharmaceutical, Osaka, Japan) was applied to the right eyes, and the corneal endothelium was recorded using a contact specular microscope (Konan Medical, Hyogo, Japan) with Scopisol (Senju Pharmaceutical, Osaka, Japan). The endothelial cell density (ECD), coefficient of variation, and hexagonal cell ratio were obtained by extracting 100 still images from the video recordings and having blinded independent evaluators randomly select three images. Analysis was performed using a previously reported U-Net convolutional neural network program for cell skeleton estimation [40]. The recorded videos were also converted to panoramic images using our previously described method [40]. Briefly, images with optimal focus were identified at approximately 0.2 s intervals using normalized roughness coefficients, generating 400 grayscale images with 480 × 720 pixel resolution. After applying Laplacian transformation to select 100 high-quality images, panoramic images were created using AutoStitch software [41]. The guttae were then identified using a deep learning program [40], and the total area ratio of guttae to the corneal area was calculated. 

### 2.13. mRNA Library Preparation and Sequencing

*Col8a2*^Q455K/Q455K^ mice (n = 3) and emricasan-treated mice (n = 3) were euthanized at 28 weeks of age, and their eyes were enucleated and immersed in RNAlater (Thermo Fisher Scientific). Under a microscope, the corneas were isolated from the eyes, and endothelial cells with Descemet membrane were carefully stripped from the corneas. The obtained tissues were immediately immersed in RNAlater and stored at 4 °C until RNA extraction. Total RNA was extracted from CECs using the RNeasy Micro Kit (QIAGEN, Hilden, Germany) according to the manufacturer’s protocol. The RNA quantity and quality were assessed using an Agilent 2100 Bioanalyzer with an RNA 6000 Pico Kit (Agilent Technologies, Santa Clara, CA, USA).

RNA sequencing libraries for next-generation sequencing (NGS) were prepared using the SMART-Seq HT Kit (Takara Bio Inc., Kusatsu, Shiga, Japan) following the manufacturer’s protocol. After full-length cDNA synthesis, Illumina libraries were prepared using NexteraXT (Illumina Inc., San Diego, CA, USA). The sequencing was performed on a NovaSeq 6000 platform (Illumina Inc.). Sequencing analysis was conducted at the Genome Information Research Center, Osaka University (Osaka, Japan).

### 2.14. RNA Sequencing Data Analysis

Raw fastq files were processed using fastp software (v0.20.4) [42] to remove low-quality reads. The processed reads were aligned with the mouse reference genome (GRCm39) using STAR (v2.7.10a) [43], and gene expression was quantified using RSEM (v1.3.1) [44]. Differential expression analysis between the vehicle group (n = 3) and the emricasan group (n = 3) was performed using DESeq2 (v1.42.1) [45]. Genes with *p*-values < 0.05 and absolute |log_2_ fold change| ≥ 1.0 were considered significantly differentially expressed. Volcano plots were generated using ggplot2 (v3.5.1) in R (v4.3.3) to visualize gene expression patterns. Protein-coding differentially expressed genes (DEGs) were identified using biomaRt (v2.58.2) [46] for downstream analyses. All raw and processed RNA-sequencing data have been deposited in the GEO database (NCBI) under accession number GSE292135.

### 2.15. Multivariate Analysis

Principal component analysis (PCA) and correlation matrix analysis were performed using R (version 4.3.0). Expression values were normalized as log_10_ (1.0 + TPM), where TPM represents transcripts per million. PCA was conducted using the prcomp function in R, while correlation analysis was performed using Spearman’s rank correlation coefficient (cor function). The correlogram was generated using the corrplot package.

### 2.16. Pathway Analysis

Gene Ontology (GO) analysis was performed using clusterProfiler (v4.10.1) and org.Mm.eg.db (v3.18.0) [47]. GO terms with *p*-values < 0.05 were considered significantly enriched and were categorized into biological process, cellular component, and molecular function. The top 10 enriched terms from each category were visualized using ggplot2. Pathway analysis was performed using the Kyoto Encyclopedia of Genes and Genomes (KEGG) database through clusterProfiler [48]. Pathways with *p*-values < 0.05 were considered significantly enriched. The top 10 enriched pathways and their corresponding gene ratios were visualized using ggplot2.

### 2.17. Statistical Analysis

The contact specular microscope measurement data are presented as box-and-whisker plots, where the boxes indicate the interquartile range (IQR), with horizontal lines representing the median and ‘+’ symbols indicating the mean. The whiskers extend to the minimum and maximum values. The qPCR data and contact specular microscope measurements were compared between the two groups using Welch’s *t*-test. Multiple group comparisons were performed using Dunnett’s test. The qPCR results are expressed as mean ± standard error of the mean (SEM). All statistical analyses were conducted using R (version 4.3.0). The multcomp library (version 1.4-25) was used for Dunnett’s multiple-comparisons test. Statistical significance was set at *p* < 0.05.

## 3. Results

### 3.1. Caspase Inhibitors Attenuate TGF-β2-Induced Apoptosis in an FECD Cell Model

**Figure 1:**
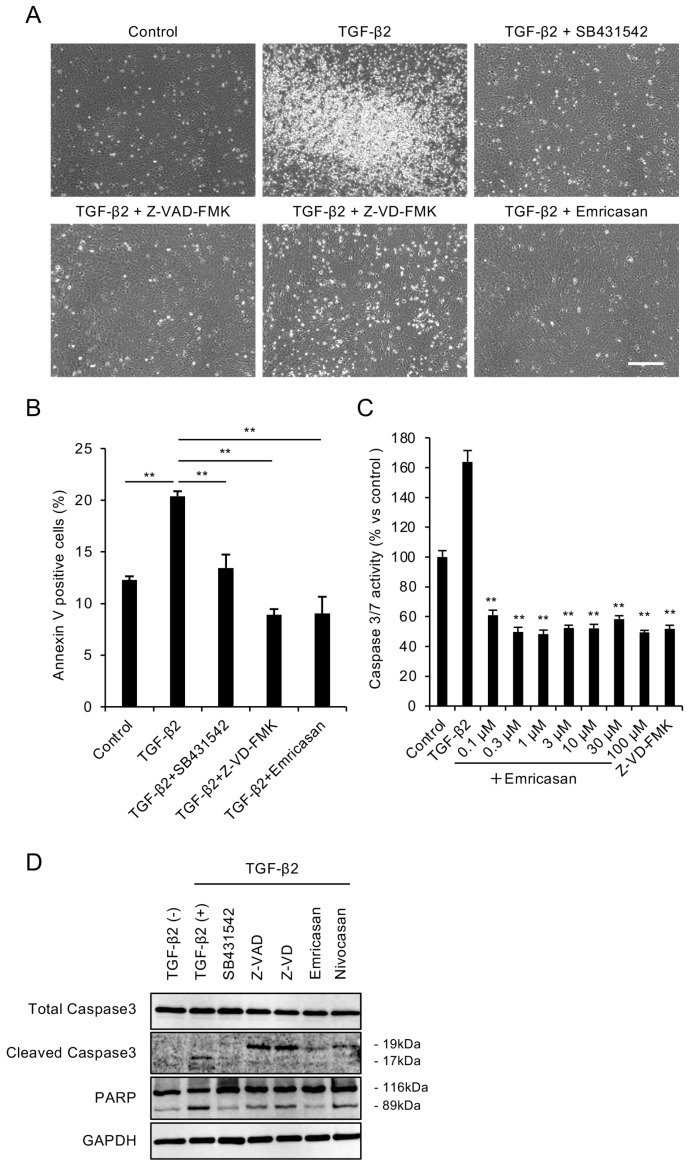
(**A**) Representative phase-contrast micrographs demonstrating that TGF-β2 (10 ng/mL, 24 h) treatment increased the number of detached cells, while cotreatment with pan-caspase inhibitors Z-VAD-FMK, Z-VD-FMK, or emricasan (all at 10 μM) markedly reduced cell detachment. Scale bar: 100 μm. Images are representative of three independent experiments. (**B**) Flow cytometry of Annexin V-positive cells. iFECD cells were treated with TGF-β2 (10 ng/mL) for 24 h in the presence or absence of SB431542, Z-VAD-FMK, Z-VD-FMK, or emricasan (all at 10 μM). TGF-β2 significantly increased the proportion of Annexin V-positive cells, but this response was attenuated by all tested inhibitors. Data represent mean ± SEM (n = 3). ** *p* < 0.01 versus TGF-β2-treated group. Representative data from two independent experiments with similar results are shown. (**C**) Effects of emricasan on TGF-β2-induced caspase-3/7 activity. The iFECD cells were exposed to TGF-β2 (10 ng/mL) with emricasan (0.1–100 μM) or Z-VD-FMK (10 μM) for 24 h. The TGF-β2-induced elevation of caspase-3/7 activity was significantly suppressed by emricasan at all concentrations tested and by Z-VD-FMK. Data represent mean ± SEM (n = 6). ** *p* < 0.01 versus TGF-β2-treated group. Data shown are representative of two independent experiments with comparable results. (**D**) Western blot analysis of apoptotic markers. Treatment with TGF-β2 induced cleavage of caspase-3 and PARP, while cotreatment with SB431542, Z-VAD-FMK, Z-VD-FMK, or emricasan inhibited this proteolytic activation. GAPDH served as a loading control. Similar results were obtained in two independent experiments; representative data are shown.

We evaluated the cytoprotective effects of the pan-caspase inhibitor emricasan in FECD by inducing TGF-β2-mediated cell death in a cell model established from an FECD patient (iFECD). To confirm that the observed cell death was mediated by TGF-β signaling activation, we utilized SB431542, a selective inhibitor of TGF-β type I receptors. We examined the effects of widely used pan-caspase inhibitors, including Z-VAD-FMK, Z-VD-FMK, and emricasan (10 μM), with the latter considered a potential therapeutic candidate for FECD treatment. Consistent with a previous report [39], phase-contrast microscopy at 24 h post-TGF-β2 exposure revealed an increase in floating dead cells in the iFECD cell culture medium. Treatment with SB431542 significantly suppressed this TGF-β2-induced increase in floating cells, indicating that cell death is mediated by TGF-β signaling activation. Treatment with Z-VAD-FMK, Z-VD-FMK, or emricasan significantly attenuated the TGF-β2-induced increase in floating cells, suggesting that caspase inhibition may protect against TGF-β2-induced cell death (Figure 1A). Annexin V assays revealed that TGF-β2 significantly increased the proportion of Annexin V-positive cells. This increase was significantly suppressed by both Z-VD-FMK and emricasan treatment, with Annexin V-positive cell rates returning to levels comparable to those of control cells (Figure 1B). 

We determined the effective concentration range of emricasan for cell death inhibition by assessing caspase-3/7 activity. TGF-β2-treated iFECD exhibited a significant 1.6-fold increase in caspase-3/7 activity. Treatment with emricasan (0.1–100 μM) suppressed this increase, maintaining caspase-3/7 activity at levels comparable to the control cells (Figure 1C). Western blot analysis demonstrated increased cleavage of caspase-3 and PARP in TGF-β2-treated iFECD cells, confirming the occurrence of apoptosis. Treatment with pan-caspase inhibitors, including Z-VAD-FMK, Z-VD-FMK, emricasan, and nivocasan, attenuated these cleavage events, indicating the suppression of apoptosis (Figure 1D).

### 3.2. Caspase Inhibitors Suppress Various Stress-Induced Apoptosis in CECs

**Figure 2:**
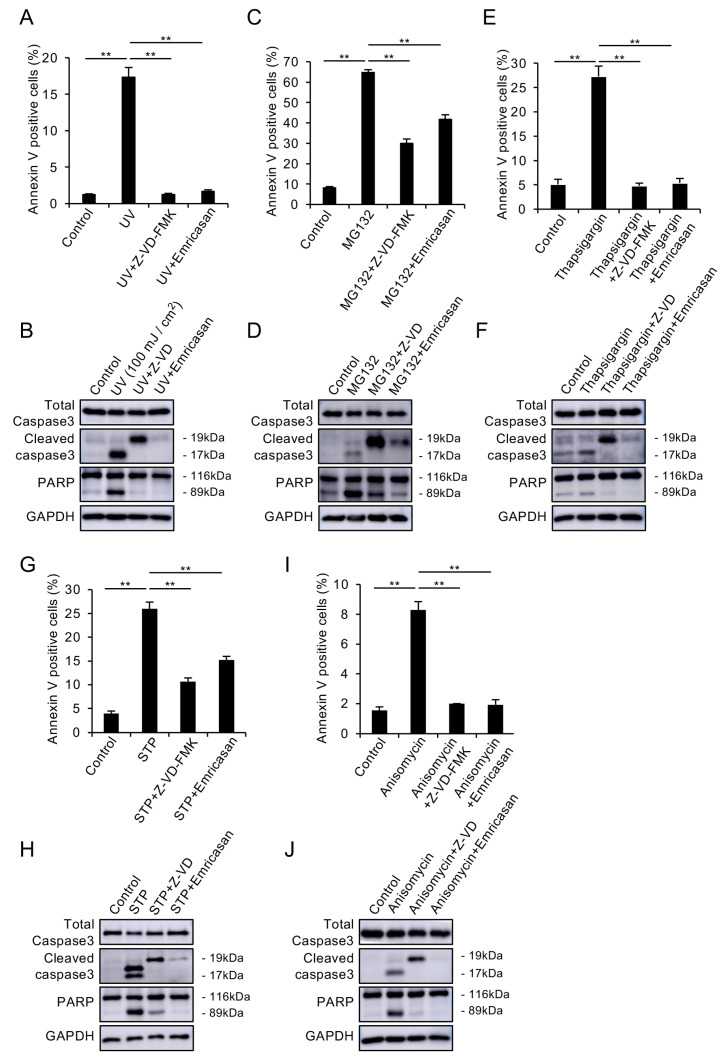
(**A**,**B**) UV-induced apoptosis model. The iHCEC cells were exposed to UV radiation (100 mJ/cm^2^) with or without Z-VD-FMK (10 μM) or emricasan (10 μM). (**A**) Flow cytometry showed that UV exposure significantly increased Annexin V-positive cells, but this response was suppressed by both inhibitors. Data represent mean ± SEM (n = 3); ** *p* < 0.01. (**B**) Western blot analysis demonstrated that UV-induced cleavage of caspase-3 and PARP was attenuated by both inhibitors. GAPDH served as a loading control. (**C**,**D**) Proteasome dysfunction model. The iHCEC cells were treated with MG132 (20 μM) ± Z-VD-FMK (10 μM) or emricasan (10 μM). (**C**) Flow cytometry revealed that MG132 significantly increased Annexin V-positive cells, but this response was prevented by both inhibitors. Data represent mean ± SEM (n = 3); ** *p* < 0.01. (**D**) Western blot analysis showed that MG132-induced caspase-3 and PARP cleavage was suppressed by both inhibitors. (**E**,**F**) ER stress model. The iHCEC cells were exposed to thapsigargin (TG, 10 μM) ± Z-VD-FMK (10 μM) or emricasan (10 μM). (**E**) Flow cytometry demonstrated that TG significantly increased Annexin V-positive cells, but this response was prevented by both inhibitors. Data represent mean ± SEM (n = 3); ** *p* < 0.01. (**F**) Western blot analysis showed that TG-induced caspase-3 and PARP cleavage was attenuated by both inhibitors. (**G**,**H**) Mitochondrial dysfunction model. The iHCEC cells were treated with staurosporine (STP, 0.5 μM) ± Z-VD-FMK (10 μM) or emricasan (10 μM). (**G**) Flow cytometry revealed that STP significantly increased Annexin V-positive cells, but this response was suppressed by both inhibitors. Data represent mean ± SEM (n = 3); ** *p* < 0.01. (**H**) Western blot analysis showed that STP-induced caspase-3 and PARP cleavage was prevented by both inhibitors. (**I**,**J**) Multiple stress pathway model. The iHCEC cells were treated with anisomycin (0.1 μM) ± Z-VD-FMK (10 μM) or emricasan (10 μM). (**I**) Flow cytometric analysis showed that anisomycin significantly increased Annexin V-positive cells, but this response was prevented by both inhibitors. Data represent mean ± SEM (n = 3); ** *p* < 0.01. (**J**) Western blot analysis demonstrated that anisomycin-induced caspase-3 and PARP cleavage was suppressed by both inhibitors. Blots are representative of at least two independent experiments.

Multiple cellular stresses, including UV exposure, endoplasmic reticulum (ER) stress, and mitochondrial dysfunction, are known to contribute to cell death in FECD [11,12,13,14,15,16]. Therefore, we evaluated the effect of emricasan on cell death induced by various stimuli that mimic these mechanisms in CECs. First, we assessed the impact of UV-induced apoptosis. In the UV-induced FECD mimic in vitro models, in which iHCEC was exposed to UV radiation (100 mJ/cm^2^), flow cytometry analysis revealed a significant increase in Annexin V-positive cells. Treatment with Z-VD-FMK or emricasan (10 μM) suppressed this increase, maintaining levels comparable to those of control cells (Figure 2A). Western blot analysis showed that UV exposure increased the cleavage of caspase-3 and PARP, which was attenuated by Z-VD-FMK and emricasan treatment (Figure 2B). We also investigated the effect on ubiquitin–proteasome system dysfunction-induced apoptosis, which is known to trigger ER stress through the accumulation of misfolded proteins. In ubiquitin–proteasome system dysfunction-induced FECD mimic models, where iHCEC cells were treated with MG132, both the proportion of Annexin V-positive cells and the cleavage of caspase-3 and PARP increased. Treatment with Z-VD-FMK or emricasan significantly attenuated these changes, although not completely to control levels (Figure 2C,D). In the ER stress-induced mimic models created by treating iHCEC cells with thapsigargin, we observed increased Annexin V-positive cells and enhanced cleavage of caspase-3 and PARP. These changes were suppressed by treatment with Z-VD-FMK or emricasan to levels comparable to control cells (Figure 2E,F). In our FECD mimic model focusing on mitochondrial dysfunction, we used staurosporine (STP), which induces disruption of mitochondrial membrane potential and cytochrome c release. When iHCEC cells were treated with STP, both the proportion of Annexin V-positive cells and the cleavage of caspase-3 and PARP increased significantly. Treatment with Z-VD-FMK or emricasan significantly reduced these changes, with Z-VD-FMK showing stronger inhibition than emricasan, though neither completely restored the values to control levels (Figure 2G,H). Treatment with anisomycin (a protein synthesis inhibitor that triggers multiple stress-response pathways, including mitochondrial dysfunction and ER stress) induced apoptosis, as indicated by greater proportions of Annexin V-positive cells and enhanced cleavage of caspase-3 and PARP. These apoptotic changes were suppressed to levels comparable to those of the control cells by treatment with Z-VD-FMK or emricasan (Figure 2I,J). These results demonstrate that caspase inhibitors effectively suppress apoptosis in CECs induced by various cellular stresses.

### 3.3. Emricasan Suppresses TGF-β2-Induced ECM Production and EMT

**Figure 3:**
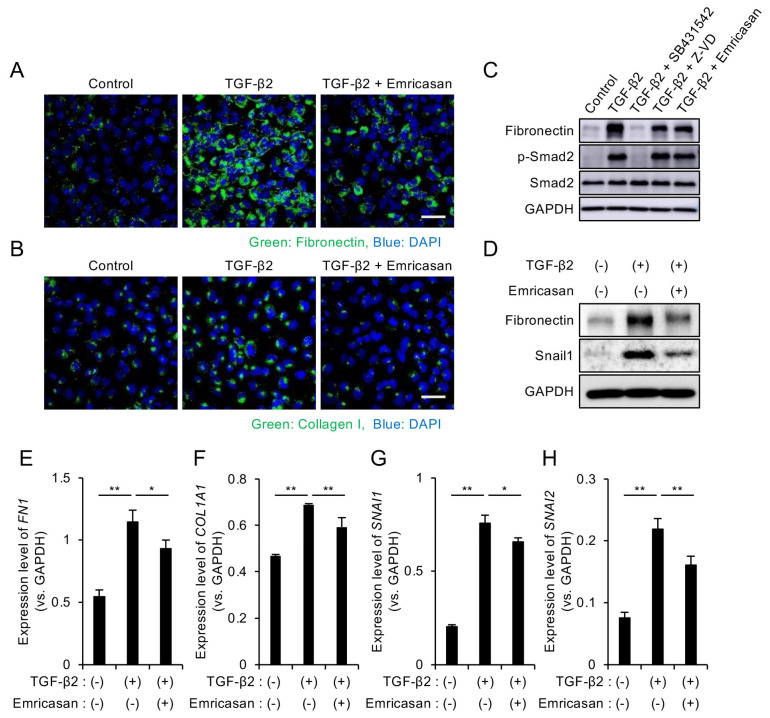
(**A**,**B**) Immunofluorescence analysis of ECM proteins. iFECD cells were treated with TGF-β2 (10 ng/mL) ± emricasan (10 μM) for 24 h. (**A**) Fibronectin and (**B**) type I collagen immunostaining (green) indicated increased expression following TGF-β2 treatment, but this response was suppressed by emricasan. Nuclei were counterstained with DAPI (blue). Scale bar: 50 μm. Images are representative of two independent experiments. (**C**,**D**) Western blot analysis of TGF-β signaling and EMT markers. (**C**) The TGF-β2-induced Smad2 phosphorylation was blocked by SB431542 (10 μM) but not by Z-VD-FMK or emricasan (10 μM). (**D**) The TGF-β2-induced upregulations of fibronectin and Snail1 were attenuated by emricasan. GAPDH served as a loading control. Blots are representative of two independent experiments. (**E**–**H**) Quantitative PCR analysis of ECM and EMT-related genes. iFECD cells were treated with TGF-β2 (10 ng/mL) ± emricasan (10 μM) for 24 h. TGF-β2 significantly increased mRNA levels of the ECM components *FN1* (**E**) and *COL1A1* (**F**) and the EMT transcription factors *SNAI1* (**G**) and *SNAI2* (**H**). Emricasan treatment significantly suppressed these transcriptional changes. Data represent mean ± SEM (n = 3). ** *p* < 0.01, * *p* < 0.05 versus TGF-β2-treated group.

We evaluated the effects of emricasan on ECM production and EMT by culturing iFECD cells with TGF-β2 in the presence or absence of emricasan for 24 h. Immunofluorescence analysis revealed that TGF-β2 treatment enhanced the expression of ECM-related proteins, fibronectin, and type I collagen, which are major components of guttae in FECD [49,50]. This enhanced expression was suppressed by emricasan treatment (Figure 3A,B). Western blot analysis demonstrated that TGF-β2 treatment induced Smad2 phosphorylation, which was inhibited by SB431542. Neither Z-VD-FMK nor emricasan suppressed Smad2 phosphorylation, indicating that the Smad-dependent (canonical) pathway remained active (Figure 3C). Consistent with the immunofluorescence results, emricasan suppressed the TGF-β2-induced increase in fibronectin expression, as shown by Western blot analysis (Figure 3D). Quantitative PCR analysis of ECM-related genes revealed that TGF-β2 treatment increased the expression of *FN1* and *COL1A1*. This increase was significantly suppressed by emricasan treatment (Figure 3E,F).

EMT is known to contribute to increased ECM production in FECD [39,51]. Therefore, we examined EMT-related markers by Western blot analysis. The TGF-β2-induced upregulation of Snail1 was suppressed by emricasan treatment (Figure 3D), and subsequent qPCR analysis confirmed that emricasan significantly reduced the TGF-β2-induced increase in *SNAIL1* and *SNAIL2* expression (Figure 3G,H).

### 3.4. Caspase-7 Knockdown Specifically Suppresses TGF-β2-Induced ECM Expression

**Figure 4:**
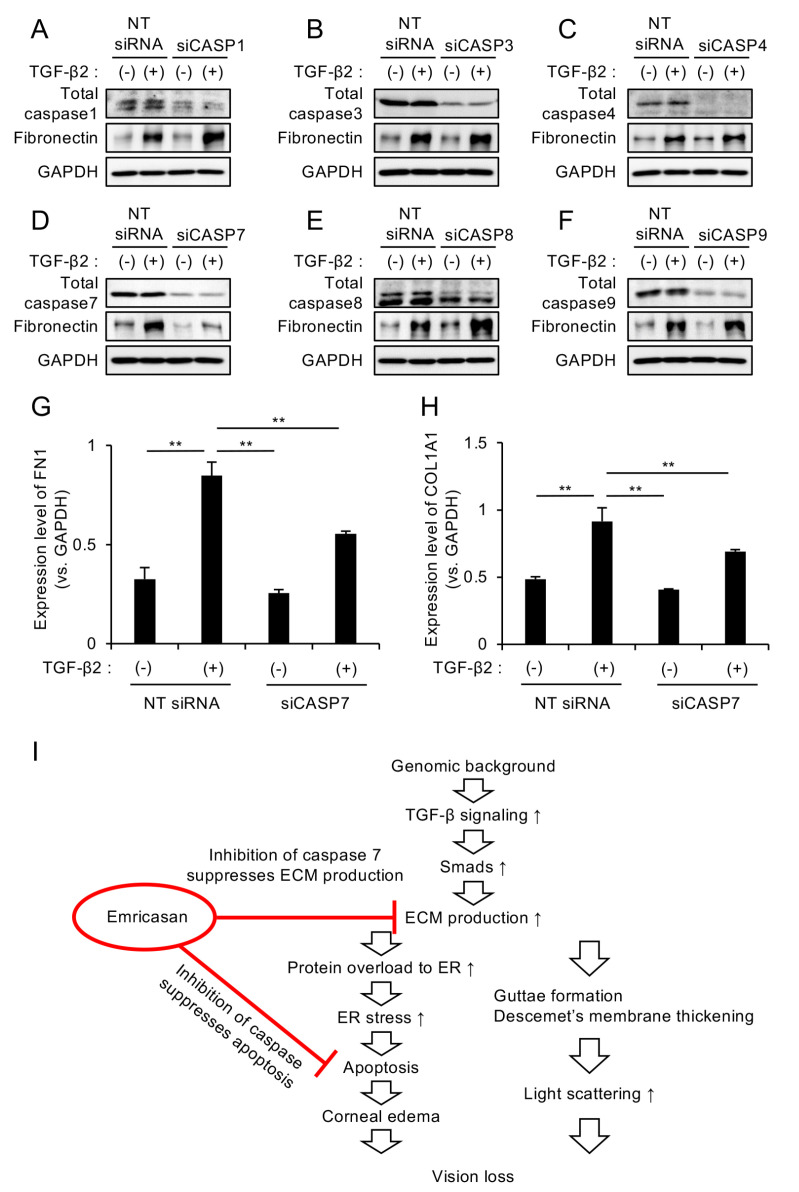
(**A**–**F**) Differential effects of individual caspase knockdown on TGF-β2-induced ECM expression. The iFECD cells were transfected with nontargeting (NT) siRNA or siRNAs targeting specific caspases, followed by TGF-β2 treatment (10 ng/mL, 24 h). Western blot analysis showed that TGF-β2-induced fibronectin upregulation was maintained in cells with caspase-1 (**A**), caspase-3 (**B**), caspase-4 (**C**), caspase-8 (**E**), or caspase-9 (**F**) knockdown. However, caspase-7 knockdown (**D**) specifically attenuated TGF-β2-induced fibronectin expression. GAPDH served as a loading control. Blots are representative of two independent experiments. (**G**,**H**) Quantitative PCR analysis of ECM components in caspase-7-depleted cells. The iFECD cells were transfected with NT siRNA or caspase-7 siRNA followed by TGF-β2 treatment (10 ng/mL, 24 h). TGF-β2-induced upregulation of *FN1* (**G**) and *COL1A1* (**H**) was significantly suppressed by caspase-7 knockdown. Data represent mean ± SEM (n = 3). ** *p* < 0.01 versus TGF-β2-treated NT siRNA group. (**I**) Schematic representation of the dual therapeutic mechanism of emricasan: specific inhibition of caspase-7-mediated ECM production and broad suppression of apoptotic pathways through pan-caspase inhibition.

Emricasan is a pan-caspase inhibitor; therefore, we sought to identify which specific caspase mediates its suppressive effect on excessive ECM production. We individually knocked down caspase-1, -3, -4, -7, -8, and -9 in iFECD using siRNA (Supplementary Figure S1) and examined the effects of these knockdowns on ECM production. TGF-β2-induced increase in fibronectin expression was observed in iFECD with caspase-1, -3, -4, -8, and -9 knockdown (Figure 4A–C,E,F). However, cells with caspase-7 knockdown showed no increase in fibronectin expression upon TGF-β2 treatment, instead maintaining levels comparable to control levels (Figure 4D).

Subsequent qPCR analysis demonstrated that caspase-7 knockdown significantly suppressed the TGF-β2-induced upregulation of the ECM-related genes *FN1* and *COL1A1*, although this suppression did not result in levels equal to those observed in the untreated control cells (Figure 4G,H). These results suggest that the pan-caspase inhibitor emricasan demonstrates a dual therapeutic effect by attenuating excessive ECM production through caspase-7 inhibition while simultaneously suppressing apoptotic pathways via its broad-spectrum caspase inhibitory activity (Figure 4I).

### 3.5. Effects of Topical Emricasan Administration on FECD Phenotype in Col8a2^Q455K/Q455K^ Mice

We evaluated the therapeutic potential of emricasan for FECD treatment by first conducting a pharmacokinetic study of topically administered emricasan. A 0.1% emricasan ophthalmic solution was developed and administered to rabbit eyes to assess its corneal penetration and distribution. High-performance liquid chromatography (HPLC) revealed emricasan concentrations of 31.2 ± 8.5 ng/mg, 5.8 ± 2.3 ng/mg, and 16.5 ± 4.5 ng/mg tissue in the corneal epithelial, stromal, and endothelial layers, respectively (Supplementary Figure S2). The endothelial concentration of 16.5 ± 4.5 ng/mg (equivalent to 29.0 µM) exceeded the effective concentration demonstrated in Figure 1C.

**Figure 5:**
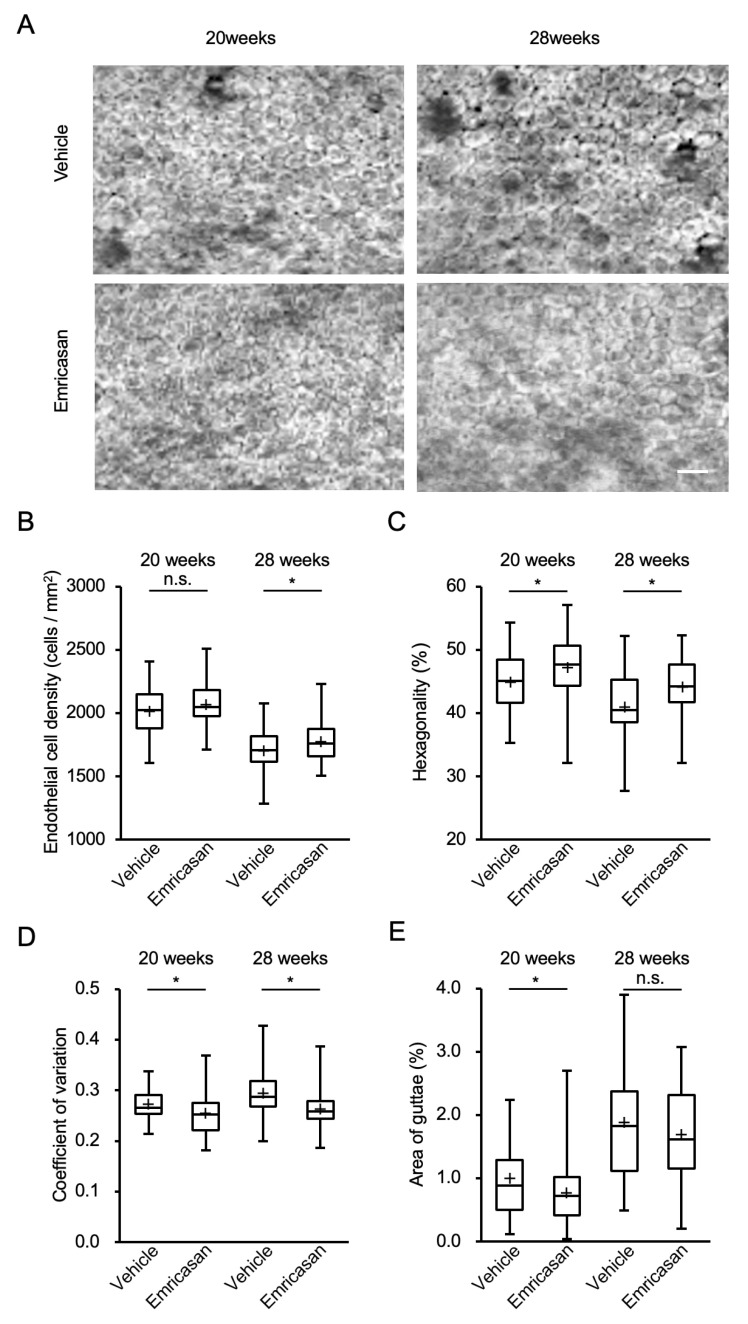
(**A**) Representative specular microscopy images of the corneal endothelium of *Col8a2*^Q455K/Q455K^ mice treated with 0.1% emricasan or vehicle eye drops twice daily from 20 to 28 weeks of age. Vehicle-treated mice exhibited progressive FECD phenotypes, including guttae formation and enlarged endothelial cells at 28 weeks. Emricasan treatment preserved endothelial cell morphology, as indicated by reduced guttae formation and higher cell density. Scale bar: 100 µm. (**B**) At 28 weeks, emricasan-treated mice maintained significantly higher ECD (1778 ± 19 cells/mm^2^) compared with vehicle-treated controls (1706 ± 15 cells/mm^2^). * *p* < 0.05. (**C**) Emricasan treatment preserved higher percentages of hexagonal cells at both 20 weeks (47.1 ± 0.6% vs. 44.9 ± 0.8%) and 28 weeks (44.0 ± 0.4% vs. 40.9 ± 1.0%). * *p* < 0.05. (**D**) Emricasan-treated corneas showed significantly lower coefficients of variation at both 20 weeks (0.255 ± 0.005 vs. 0.272 ± 0.005) and 28 weeks (0.262 ± 0.004 vs. 0.293 ± 0.008). * *p* < 0.05. (**E**) At 28 weeks, emricasan treatment significantly reduced the guttae area percentage (0.75 ± 0.05%) compared with vehicle treatment (1.00 ± 0.09%). * *p* < 0.05. For all analyses (**B**–**E**), Col8a2Q455K/Q455K mice received either 0.1% emricasan eye drops (emricasan group, n = 68) or vehicle eye drops (vehicle group, n = 35) administered as 2 µL twice daily in the right eye from 8 to 28 weeks of age.

Based on these findings, we investigated the effects of twice-daily administration of 0.1% emricasan eye drops in *Col8a2*^Q455K/Q455K^ mice from 8 to 28 weeks of age. Contact specular microscopy revealed that the vehicle-treated group exhibited characteristic FECD phenotypes, including guttae formation and enlarged endothelial cells with decreased cell density, with these features becoming more pronounced at 28 weeks of age. By contrast, the emricasan-treated group showed reduced guttae formation and smaller endothelial cells with higher cell density compared with the vehicle group (Figure 5A). ECD analysis at 20 weeks showed a trend toward higher values in the emricasan group (n = 68) compared with the vehicle group (n = 35) (2067 ± 19 cells/mm^2^ vs. 2019 ± 32 cells/mm^2^, respectively; *p* = 0.200). At 28 weeks, the emricasan group demonstrated significantly higher ECD compared with the vehicle group (1778 ± 19 cells/mm^2^ vs. 1706 ± 15 cells/mm^2^, respectively; *p* = 0.048) (Figure 5B). Morphological analysis revealed significantly better-preserved hexagonality in the emricasan group at both 20 weeks (47.1 ± 0.6% vs. 44.9 ± 0.8%; *p* = 0.034) and 28 weeks of age (44.0 ± 0.4% vs. 40.9 ± 1.0%; *p* = 0.007) (Figure 5C). The coefficient of variation was significantly lower in the emricasan group at both time points (20 weeks: 0.255 ± 0.005 vs. 0.272 ± 0.005, *p* = 0.017; 28 weeks: 0.262 ± 0.004 vs. 0.293 ± 0.008, *p* = 0.001) (Figure 5D). Quantification of guttae area showed significantly lower values in the emricasan group at 20 weeks compared with the vehicle group (0.75 ± 0.05% vs. 1.00 ± 0.09%, respectively; *p* = 0.031). At 28 weeks, although the emricasan group showed a trend toward a smaller guttae area, the difference did not reach statistical significance (1.68 ± 0.09% vs. 1.89 ± 0.15%, respectively; *p* = 0.249) (Figure 5E).

### 3.6. Effects of Topical Emricasan Administration on Gene Expression in Corneal Endothelium of Col8a2^Q455K/Q455K^ Mice

**Figure 6:**
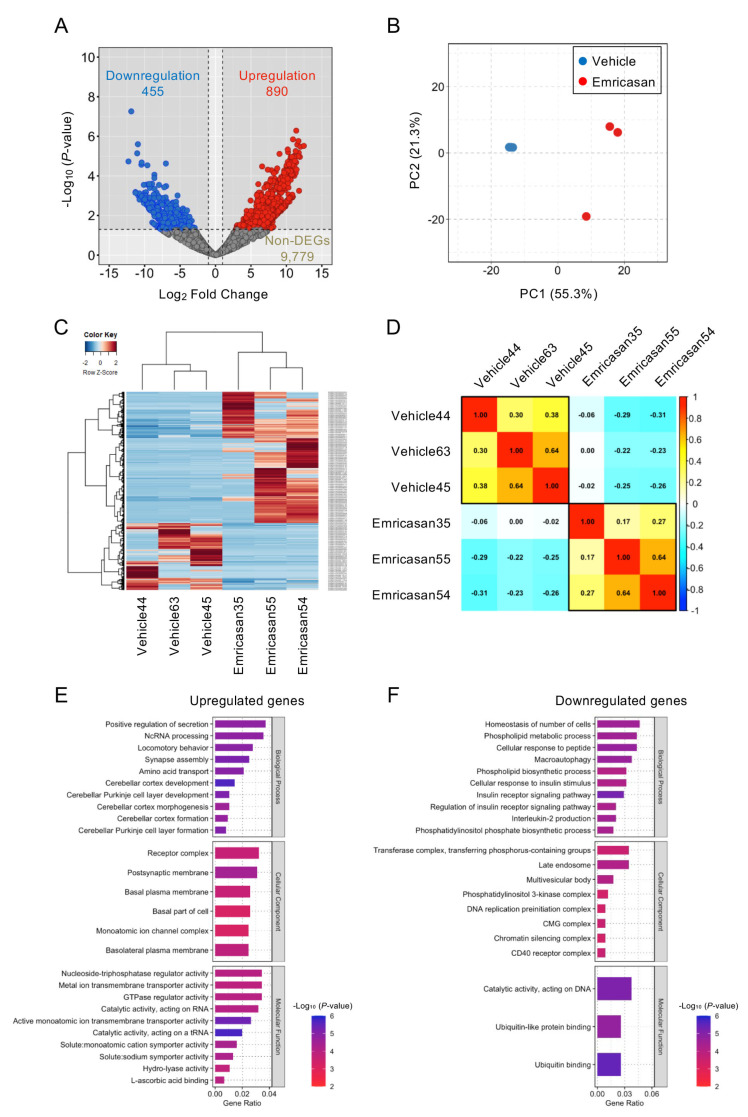
(**A**) Differential gene expression analysis of corneal endothelium from *Col8a2*^Q455K/Q455K^ mice treated with 0.1% emricasan or vehicle eye drops twice daily (20–28 weeks of age; n = 3 per group). Volcano plot depicts 890 upregulated (red) and 455 downregulated (blue) genes among 11,124 expressed genes in the emricasan group. *X*-axis: log2 fold change; *Y*-axis: −log10 (*p*-value). (**B**) Principal component analysis demonstrating distinct clustering of vehicle-treated (blue) and emricasan-treated (red) samples. The percentage of variance explained by PC1 and PC2 is indicated on the respective axes. (**C**) Hierarchical clustering heatmap of differentially expressed genes between the vehicle and emricasan groups. Color intensity indicates the relative expression levels (red: high, blue: low). Groups and genes are indicated at the bottom and right, respectively. (**D**) Spearman’s rank correlation matrix with hierarchical clustering (Ward’s method) showing clear separation between treatment groups. (**E**) Top-enriched Gene Ontology (GO) terms among upregulated genes ranked by statistical significance. *X*-axis indicates the proportion of altered genes within each GO category. (**F**) Top-enriched GO terms among downregulated genes ranked by statistical significance. *X*-axis indicates the proportion of altered genes within each GO category.

RNA sequencing analysis identified 1345 DEGs among 11,124 total expressed genes in *Col8a2*^Q455K/Q455K^ mice compared with emricasan-treated *Col8a2*^Q455K/Q455K^ mice (455 upregulated and 890 downregulated genes; fold change ≥ 1.5, adjusted *p* < 0.05) (Figure 6A). PCA revealed distinct transcriptional profiles between the groups, with clear separation along the first principal component (Figure 6B). Hierarchical clustering analysis of the DEGs demonstrated distinct expression patterns between the *Col8a2*^Q455K/Q455K^ and emricasan-treated groups, visualized in a heatmap showing clear segregation of samples by treatment group (Figure 6C). Pearson correlation analysis demonstrated high intra-group similarity (correlation coefficients: 0.30–0.64 for *Col8a2*^Q455K/Q455K^ mice; 0.17–0.64 for emricasan-treated mice) and lower inter-group correlation (−0.31 to 0.00), confirming distinct transcriptional signatures between the groups (Figure 6D).

GO enrichment analysis revealed that 43 biological processes, 8 cellular components, and 3 molecular function terms were significantly enriched among the DEGs (*p*-value < 0.05). Among the upregulated genes, enriched terms included positive regulation of secretion, ncRNA processing, and locomotory behavior (biological processes); receptor complex, postsynaptic membrane, and basal plasma membrane (cellular components); and nucleoside-triphosphatase regulator activity, metal ion transmembrane transporter activity, and GTPase regulator activity (molecular functions) (Figure 6E). The downregulated genes showed enrichment in terms related to cellular number homeostasis, phospholipid metabolic processes, and cellular response to peptide (biological processes); transferase complex, late endosome, and multivesicular body (cellular components); and catalytic activity on DNA, ubiquitin-like protein binding, and ubiquitin binding (molecular functions) (Figure 6F).

## 4. Discussion

In the present study, we investigated the cytoprotective effects of emricasan in FECD using multiple disease models established from FECD patient-derived cells as well as in various cellular injury models that mimicked FECD pathology through UV exposure, ER stress, and mitochondrial dysfunction [1,2,3]. We also revealed that only caspase-7, among other caspase types, plays a crucial role in ECM suppression. Furthermore, we demonstrated the therapeutic potential of emricasan as a form of eye drop through in vivo studies using an FECD mouse model. 

The pan-caspase inhibitor emricasan has demonstrated the clear therapeutic potential of apoptosis modulation, as confirmed by extensive investigations in various disease contexts [52,53,54,55]. Clinical trials have revealed its efficacy in liver diseases, where it has shown reductions in portal hypertension in cirrhosis patients and decreased caspase activation in non-alcoholic fatty liver disease [29,30,32]. While larger trials have produced variable outcomes across different hepatic conditions [31,33], multiple clinical investigations, including recent Phase 1 trials for COVID-19 treatment (NCT04803227), have established a comprehensive safety profile for emricasan. For this reason, and because of its availability among clinically investigated caspase inhibitors, we selected emricasan to investigate its therapeutic potential in FECD.

Accumulating evidence indicates that apoptosis plays a fundamental role in the pathogenesis of FECD. Previous research involving TUNEL assays has demonstrated significantly increased apoptotic activity in FECD corneal endothelium relative to control specimens [11,56]. Additional immunohistochemical analyses have revealed upregulation of pro-apoptotic markers, specifically Fas, FasL, and Bax, in FECD tissue samples [56,57]. Analysis at the molecular level has identified elevated activation of apoptotic mediators, notably caspase-9 and caspase-3, in FECD endothelial cells [13]. Ultrastructural examination by transmission electron microscopy has documented mitochondrial fragmentation and cytochrome c release [58]. Biochemical analyses have demonstrated p53 pathway activation in FECD, characterized by an increased accumulation of phosphorylated p53 [12]. Furthermore, mechanistic investigations have established that FECD cells show increased vulnerability to oxidative stress-mediated apoptosis via p53-dependent pathways [12,59,60]. The present study advances these findings through our in vivo demonstration that apoptosis inhibition represents a viable therapeutic strategy in an FECD mouse model. This investigation provides the first experimental validation of apoptosis suppression as a therapeutic approach in a living system, confirming both the pathogenic role of apoptosis in FECD and establishing a foundation for novel therapeutic interventions.

A principal limitation of this study is the incomplete elucidation of the molecular mechanism by which emricasan suppresses excessive ECM production in FECD. Though our data demonstrate that pan-caspase inhibition significantly reduces apoptotic markers in FECD models, it should be noted that other regulated cell death pathways such as caspase-independent apoptosis, necroptosis, or ferroptosis may contribute to endothelial cell loss and disease progression. Nevertheless, our investigation revealed that among the various caspases inhibited by this pan-caspase inhibitor, specific suppression of caspase-7 results in reduced ECM production. This finding is particularly noteworthy in light of previous research by Ke et al., who demonstrated caspase-7 activation in renal tubulointerstitial fibrosis and TGF-β1-induced trans differentiation of tubular epithelial cells [61]. The identification of caspase-7 as a key mediator of ECM overproduction in FECD, analogous to its role in renal fibrosis, represents a novel finding in FECD pathobiology and warrants further investigation.

While the exact mechanism by which caspase-7 promotes ECM expression requires further investigation, several possibilities exist based on current evidence. Caspase-7, beyond its traditional role in apoptosis, likely influences ECM regulation through non-apoptotic functions, as demonstrated by our findings. This is consistent with the growing body of evidence showing that caspase-7 participates in diverse cellular processes beyond cell death [62,63,64], including matrix interactions. Potential mechanisms include the caspase-7-mediated cleavage of transcriptional regulators that normally suppress ECM genes or the indirect enhancement of TGF-β signaling through non-canonical pathways. Caspase-7 might also affect the activity or localization of transcription factors involved in ECM regulation, such as those in the Smad family, independent of canonical signaling events. Understanding these mechanisms could provide new therapeutic targets for addressing the pathological ECM accumulation characteristic of FECD.

## 5. Conclusions

In conclusion, this study demonstrates that emricasan exhibits therapeutic potential as an ophthalmic solution for FECD treatment while more broadly establishing the efficacy of caspase inhibitors as a therapeutic class for this disease. Our preclinical evidence supports the development of topical caspase inhibitors, particularly emricasan, as potential pharmacological interventions for FECD. Furthermore, the identification of a specific role for caspase-7 in ECM regulation provides a new direction for targeted therapeutic development in FECD treatment.

## Data Availability

Data are contained within the article or Supplementary Materials.

## Supplementary Materials

The following supporting information can be downloaded at: https://www.mdpi.com/article/10.3390/cells14070498/s1, Figure S1: Validation of individual caspase-1, -3, -4, -7, -8, and-9 knockdown by siRNA; Figure S2: Pharmacokinetic study of topically administered 0.1% emricasan in rabbit eyes.
