# Supplementary material for: Therapeutic Potential of Emricasan, a Pan-Caspase Inhibitor, in Reducing Cell Death and Extracellular Matrix Accumulation in Fuchs Endothelial Corneal Dystrophy
Source: Cells. 2025 Mar 27;14(7):498. doi: 10.3390/cells14070498 (PMC11988121; doi:10.3390/cells14070498)
Supplement: Supplementary file 1 [file cells-14-00498-s001.zip › cells-3532487-supplementary.pdf]

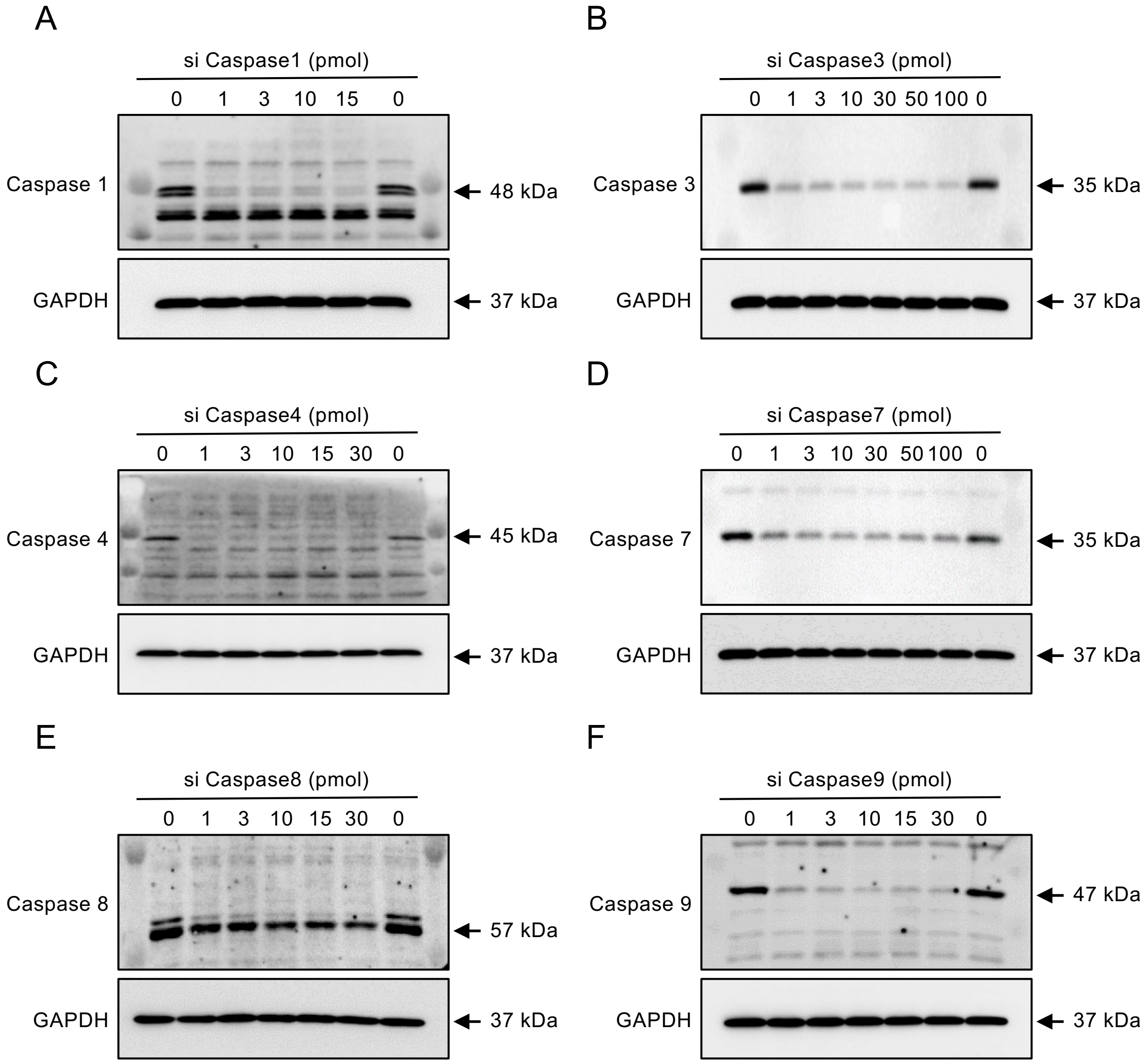

Supplementary Figure S1. Validation of Individual Caspase-1, -3, -4, -7, -8, and -9 Knockdown by siRNA

Caspase-1, -3, -4, -7, -8, and -9 were individually knocked down in iFECD cells using specific siRNAs at concentrations of 0, 1, 3, 10, 15, and 30 pmol. Western blot analysis was performed to confirm the knockdown efficiency of Caspase-1 (A), -3 (B), -4 (C), -7 (D), -8 (E), and -9 (F).

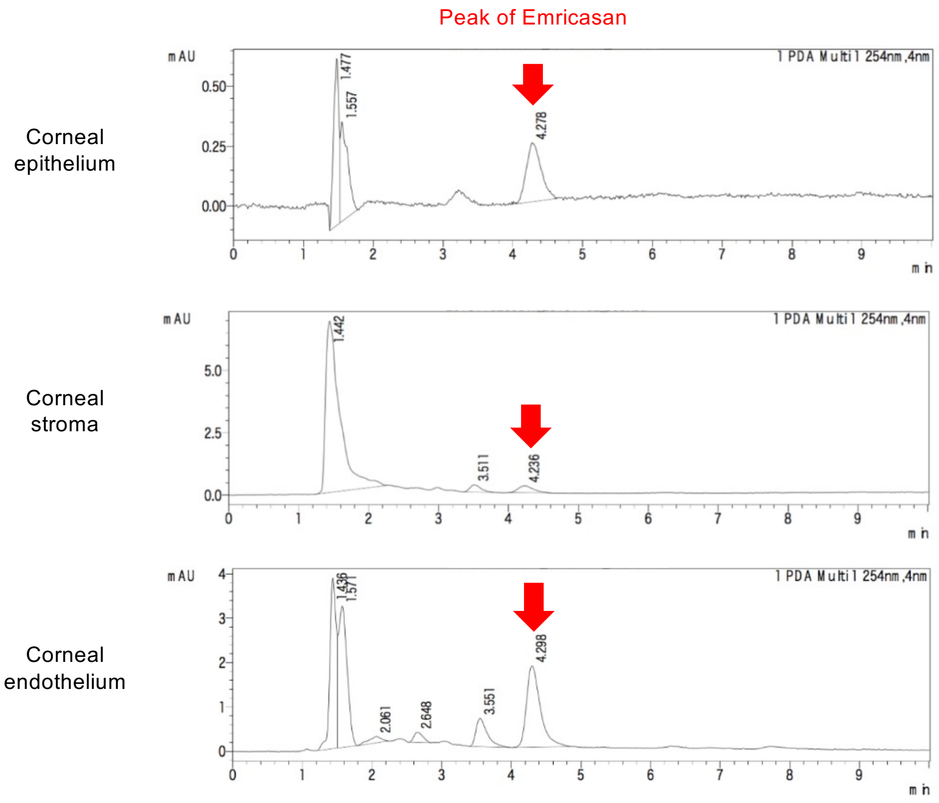

Supplementary Figure S2. Pharmacokinetic Study of Topically Administered 0.1% Emricasan in Rabbit Eyes

Slc:JW/CSK rabbits (n=3) received a single topical administration of 0.1% emricasan ophthalmic solution (50 μL) in the right eye. Following euthanasia at 1 hour post-administration, the eyes were enucleated and the corneal tissue was harvested. The cornea was mechanically dissected into epithelial, stromal, and endothelial layers, and each layer was weighed independently. High-performance liquid chromatography (HPLC) analysis revealed quantifiable emricasan concentrations in all corneal layers. The mean tissue concentrations (± standard deviation) were 31.2 ± 8.5 ng/mg in the epithelium, 5.8 ± 2.3 ng/mg in the stroma, and 16.5 ± 4.5 ng/mg in the endothelium. These findings demonstrate that topically administered emricasan successfully penetrates through the corneal tissue to reach the corneal endothelium.
